# *Telfairia occidentalis* stimulates hepatic glycolysis and pyruvate production via insulin-dependent and insulin-independent mechanisms

**DOI:** 10.1016/j.metop.2021.100092

**Published:** 2021-04-14

**Authors:** Toyin Mohammed Salman, Mayowa Adewale Iyanda, Abdul-Musawwir Alli-oluwafuyi, Sheu Oluwadare Sulaiman, Abdullateef Isiaka Alagbonsi

**Affiliations:** a Department of Physiology, College of Health Sciences, University of Ilorin, Ilorin, Nigeria; b Department of Pharmacology and Therapeutics, College of Health Sciences, University of Ilorin, Ilorin, Nigeria; c Physiology Department, Kampala International University - Western Campus, Ishaka-Bushenyi, Uganda; d Department of Morphology (Cell Biology), Instituto de Ciências Biológicas, Universidade Federal de Minas Gerais, Belo Horizonte, Brazil; e Department of Clinical Biology (Physiology), School of Medicine and Pharmacy, College of Medicine and Health Sciences, University of Rwanda, Huye Campus, Rwanda

**Keywords:** Plasma glucose, *Telfairia occidentalis*, Insulin, Glucoregulatory enzymes, Glycogen, Glucose metabolites, TO, *Telfairia occidentalis*, ATP, Adenosine triphosphate, EATO, Ethyl acetate TO fraction, NHTO, N-hexane TO fraction, NAD, Nicotinamide adenine dinucleotide, GCK, Glucokinase, ELISA, Enzyme-linked immunosorbent assay, G6Pase, Glucose-6-phosphatase, HRP, Horseradish Peroxidase, G6PD, Glucose-6-phosphate dehydrogenase, LDH, Lactate dehydrogenase, G6P, Glucose-6-phosphate, HClO_4_, Perchloric acid, KOH, Potassium hydroxide, SEM, Standard error of mean, IMGU, Insulin-mediated glucose uptake, GSIS, glucose-stimulated insulin secretion, GLUT, Glucose transporter, ANOVA, Analysis of Variance, MCT, Monocarboxylate transporters, TCA, Tricarboxylic acid cycle

## Abstract

**Background:**

*Telfairia occidentalis (TO)*, a plant consumed for its nutritional and medicinal values, exhibits hypoglycaemic effect. However, the metabolic fate of the glucose following TO-induced insulin secretion and consequent hypoglycaemia is not clear.

**Objective:**

This study determined the effect of ethyl acetate and n-hexane fractions of TO leaf extracts on some biochemical parameters in the glucose metabolic pathway to explain the possible fate of blood glucose following TO-induced hypoglycaemia.

**Methods:**

Eighteen male Wistar rats (180–200 g) divided into control, n-hexane TO fraction- and ethyl acetate TO fraction-treated groups (n = 6/group) were used. The control animals received normal saline while the treated groups received TO at 100 mg/kg for seven days. After 24 h following the last dose, the animals were anaesthetised using ketamine; blood samples were collected and livers harvested to determine some biochemical parameters.

**Results:**

Ethyl acetate TO fraction significantly increased plasma insulin, liver glucokinase activity and plasma pyruvate concentration, but significantly decreased plasma glucose and liver glycogen, without significant changes in plasma lactate, glucose-6-phosphate, liver glucose-6-phosphatase and lactate dehydrogenase activities when compared with control. N-hexane TO fraction significantly reduced liver glucose-6-phosphatase activity and glycogen but significantly increased plasma pyruvate, without significant changes in plasma glucose, insulin, glucose-6-phosphate and lactate concentrations; and liver glucokinase and lactate dehydrogenase activities.

**Conclusion:**

The present study showed that insulin-mediated TO-induced hypoglycaemia resulted in the stimulation of glycolysis and pyruvate production via insulin-dependent and insulin-independent mechanisms.

## 1 Introduction

*Telfairia occidentalis*, also called fluted gourd or pumpkin, is an ever-green food crop widely cultivated in many West African regions, mainly in Sierra Leone and South East/South-South parts of Nigeria for its nutritional and medicinal uses. The leaves and seeds are the most widely consumed parts as vegetable and oil source, respectively, and the plant’s pharmacological activities have been extensively reviewed [1]. Some pharmacological activities of TO leaves and seeds include anti-plasmodial [2], antihypertensive [3], anti-anaemic [4], and antioxidant [5,6] activities. While the leaves are a rich source of carbohydrates (sucrose), ascorbic acid, and polyphenols, the seeds have high beta-globulin proteins and oils, mainly monounsaturated fatty acids [7].

The hypoglycaemic effect of TO leaf extracts has been well-documented [[8], [9], [10], [11]]. A single administration of TO leaf extracts acutely reduced fasting blood glucose in fasted alloxan-induced diabetic animals [8,9], while the repeated administration reduced fasting blood glucose in normoglycaemic animals [11,12] and diabetic animals [13].

Our previous studies [12,14,15] have attributed the blood-glucose-lowering effect of to the increase in plasma insulin following treatment with TO. However, the appropriate question is, what was the fate of the glucose transported from the blood under insulin’s influence in the TO-treated animals? Was it phosphorylated and converted to glycogen for storage or converted to pyruvate and oxidised for ATP/energy production? The liver is an essential organ in glucose homeostasis and metabolism, capable of storing glucose as glycogen after feeding and releasing glucose from the stored glycogen between meals. Thus, liver glycogen was measured in our previous study [14] with the assumption that the glucose was converted to glycogen. Interestingly, rather than increase, the liver glycogen decreased in the study’s treated groups. Also, the plasma lactate reduced, thus ruling out the possibility of the glucose being converted to lactate or glycogen. However, the study was limited by the absence of data on the plasma pyruvate and liver glucokinase activity, which rendered the information on the fate of glucose inconclusive.

Like other solvents, n-hexane and ethyl acetate fractions of plant extracts are being used in biological experiments to study plant bioactive components’ effects because the two solvents have different polarities and can dissolve different plant bioactive components [16,17]. Thus, plant extracts of the two solvents have different biological effects depending on the fractions’ bioactive components. Besides, a single 250 mg/kg dose of ethyl acetate TO fraction had a significant hypoglycaemic effect in diabetic rats [9].

Despite the well-documented hypoglycaemic effect of TO, little or no attempt has been made to determine the hypoglycaemic effect of different TO fractions or elucidate the fate of glucose following TO-induced hypoglycaemia. The present study was designed to investigate the metabolism of glucose in the glycolytic pathway using ethyl acetate (EATO) and n-hexane (NHTO) fractions of TO with a view to ascertaining the exact end product of glucose metabolism following TO-induced hypoglycaemia.

## 2 Materials and methods

### 2.1 Animals

We used Male Wistar albino rats (180–200 g) obtained from the Animal House of the College of Health Sciences, University of Ilorin. The rats were housed in wire mesh cages under optimal temperature (25–29 °C), regular 12/12-h light/dark cycle, and *ad libitum* access to standard rat pelletised chow and water. Animals were acclimatised for two weeks before enrolling them in the study. Our institutional research and the ethical committee approved the study protocol and animal use before the study. Animal care and experimental procedures were performed following recommendations from Helsinki’s declaration on guiding principles in the care and use of animals.

### 2.2 Plant material, preparation, and extraction

Fresh leaves of TO were bought at Oja Tuntun, Ilorin, Kwara State, Nigeria. Mr Bolu Ajayi of the Department of Plant Biology, University of Ilorin, Ilorin, Nigeria authenticated the plant and the voucher number (UIH1063) was deposited in the departmental herbarium. The leaves were thoroughly but gently washed under running tap water and air-dried until a constant weight of 4.96 kg was obtained. The dried leaves were then ground into powder using a grinding machine. Five hundred grams (500 g) of the ground leaf was soaked with 4 L of 96% ethanol for three days at room temperature and shaken periodically. On the third day, the mixture was filtered and the filtrate concentrated using a rotary evaporator. The evaporated extract was dried in a desiccator containing silica gel to obtain solid crude ethanolic extract. Fifty grams (50 g) of the ethanolic crude extract was fractionated by successively adding 250 ml of each of n-hexane and ethyl acetate in increasing order of polarities to obtain n-hexane and ethyl acetate fractions. After getting each fraction, each solvent was evaporated by rotary evaporator and 18.2 g of n-hexane and 20.9 g of ethyl acetate solid fractions were obtained [9,16]. All extracts were preserved at 4 °C and dissolved in normal saline during administration.

### 2.3 Experimental procedures

Eighteen (18) male Wistar rats (180–200 g) were divided into three groups (n = 6/group) of control (received 2 ml/kg normal saline), ethyl acetate TO fraction (EATO, received 100 mg/kg of ethyl acetate fraction of TO)-, and n-hexane TO fraction (NHTO, received 100 mg/kg of n-hexane fraction of TO)-treated groups. The administration was done orally for seven days.

### 2.4 Sample collection

Twenty-four hours after the last treatment, rats were anaesthetised by administering 0.2 ml/kg of ketamine hydrochloride and killed by cervical dislocation. Blood samples were then collected via cardiac puncture into heparinised bottles and centrifuged at 3000 rpm for 15 min. Plasma was then collected into plain bottles and refrigerated at 4 °C for biochemical analyses. The liver was also collected, rinsed in ice-cold PBS solution (Sigma-Aldrich, Saint-Louis, MO, USA) three times, dried and weighed. Then, 0.5 g of the liver was weighed and chopped before being transferred into a tissue grinder and homogenised in 4% sucrose solution. The homogenates were stored at 4 °C and later used for liver glycogen and enzyme assays.

### 2.5 Assessment of parameters

#### 2.5.1 Plasma glucose

Plasma glucose was determined spectrophotometrically using assay kits as previously described [15] and based on the glucose oxidase method [18].

#### 2.5.2 Plasma insulin

Enzyme-linked immunosorbent assay (ELISA) kit (Calbiotech, USA; Catalog number: IN374S) was used to assay plasma insulin and Beckman Coulter DTX 880 Multimode Detector was used to take spectrophotometric readings following manufacturer’s protocols of the assay kits.

#### 2.5.3 Liver glycogen

The liver glycogen was assayed as described [14,19].

#### 2.5.4 Liver enzymes activity

Glucokinase (GCK) and Glucose-6-phosphatase (G6Pase) activities assays were carried out as we previously described [15].

Lactate dehydrogenase (LDH) assay was done spectrophotometrically with LDH kit (MTD diagnostics with reference number CC1256).

#### 2.5.5 Plasma concentrations of glucose metabolites

G6P was determined fluorimetrically as earlier described [20]. Pyruvate (Elabscience Biotechnology Co. Ltd, China; Catalog No: E-BC-K130) and lactate (Fortress diagnostics, UK; Product code: BXC0621) kits were used for the determination of plasma pyruvate and lactate concentrations. Spectrophotometric method was used with Beckman Coulter DTX 880 Multimode Detector).

### 2.6 Data and statistical analysis

All analyses were performed using GraphPad Prism 8 for Windows (GraphPad Software 2365 Northside Dr. Suite 560 San Diego, CA 92108) and values were expressed as mean ± SEM. One-way Analysis of Variance (ANOVA) was used to compare changes between groups, followed by a posthoc Bonferroni’s multiple comparisons test. Values were considered significant at p **<** 0.05.

## 3 Results

### 3.1 Ethyl acetate fraction of Telfairia occidentalis reduced fasting plasma glucose

**Fig. 1:**
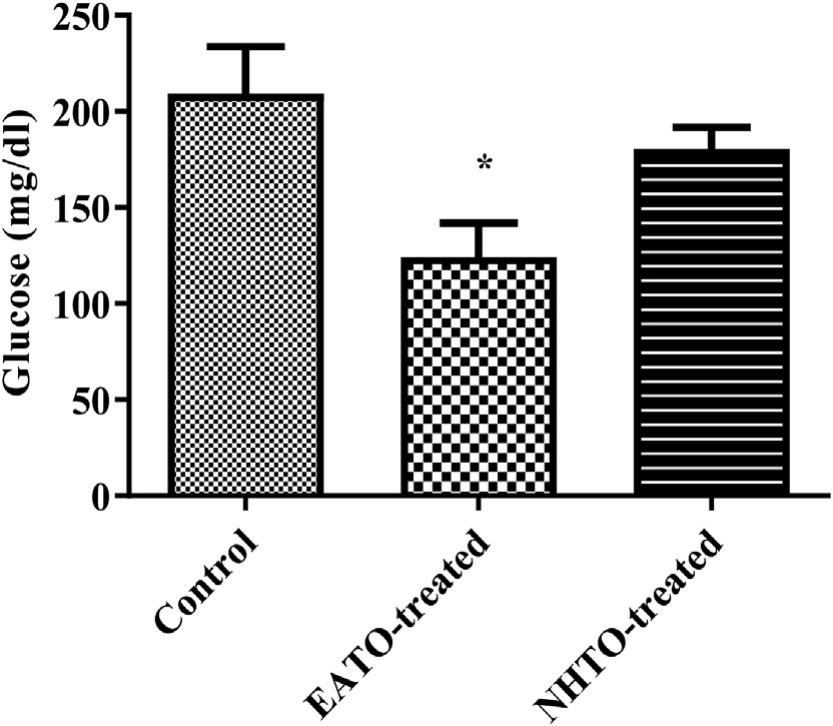
**Effects of fractions of *Telfairia occidentalis* on plasma glucose.** ∗P < 0.05 vs control. EATO: Ethyl acetate TO fraction; NHTO; N-hexane TO fraction.

Compared to the control, EATO significantly (p < 0.05) decreased plasma glucose (from 232.00 ± 12.94 mg/dl to 123.30 ± 17.64 mg/dl) while NHTO (180.7 ± 11.11 mg/dl) did not (Fig. 1).

### 3.2 Ethyl acetate fraction of Telfairia occidentalis increased plasma insulin level

**Fig. 2:**
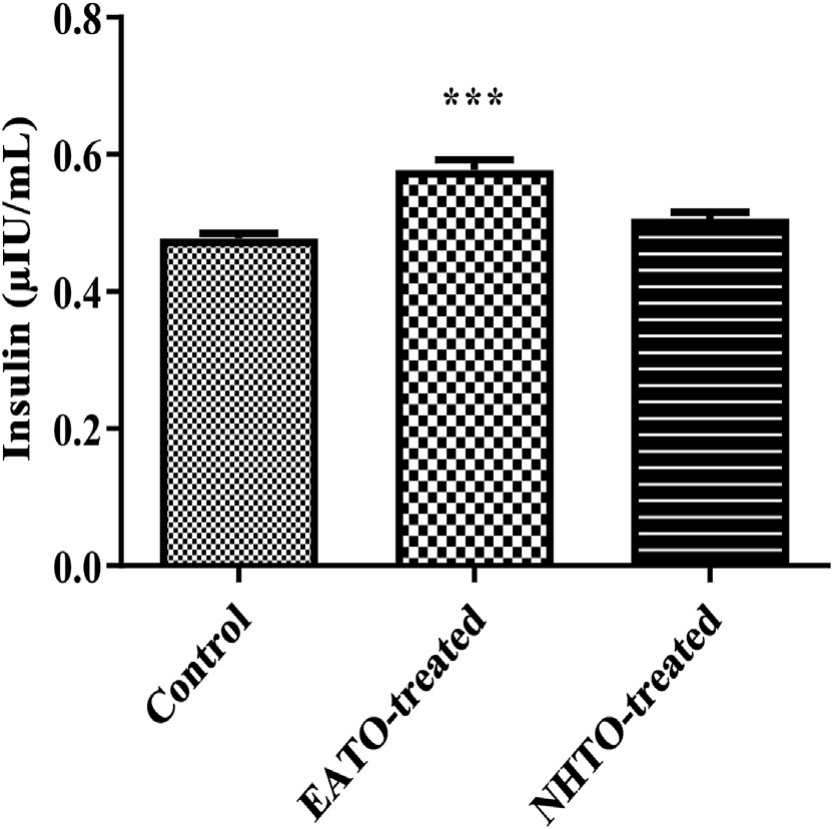
**Effects of fractions of *Telfairia occidentalis* on plasma insulin.** ∗∗∗P < 0.001 vs control. EATO: Ethyl acetate TO fraction; NHTO; N-hexane TO fraction.

EATO significantly (p < 0.001) increased plasma insulin (from 0.48 ± 0.01 μIU/ml to 0.58 ± 0.01μIU/ml), while NHTO (0.51 ± 0.01μIU/ml) did not (Fig. 2).

### 3.3 EATO and NHTO reduced liver glycogen concentration

**Fig. 3:**
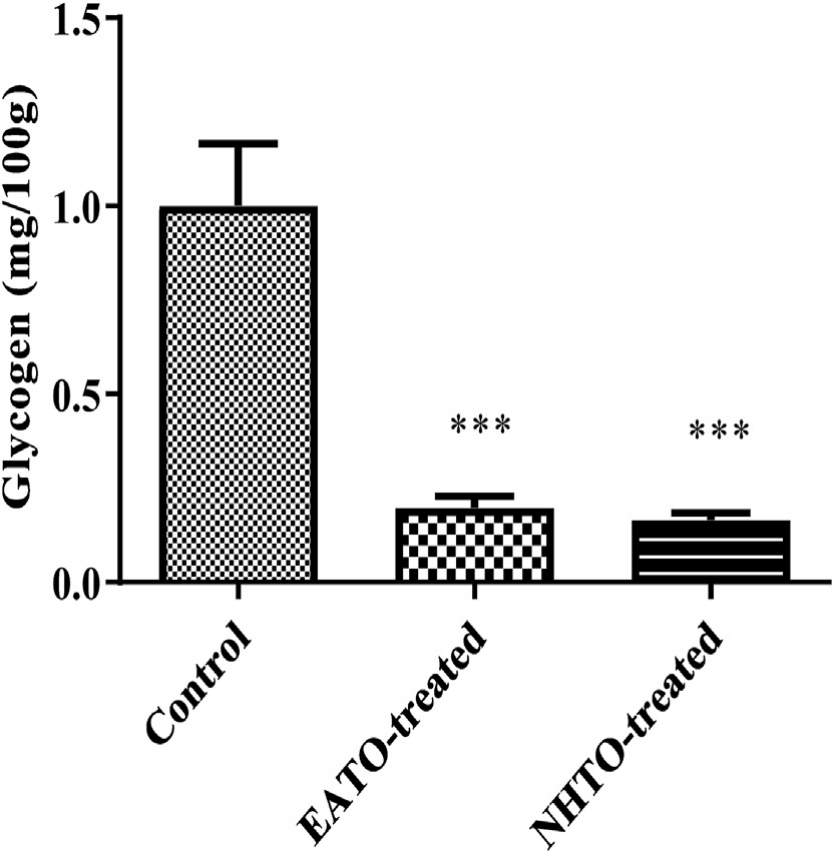
**Effects of fractions of *Telfairia occidentalis* on liver glycogen concentration.** ∗∗∗P < 0.001 vs control. EATO: Ethyl acetate TO fraction; NHTO; N-hexane TO fraction.

There were significant decreases (P < 0.001) in liver glycogen concentrations in rats treated with both EATO (0.20 ± 0.03 mg/100 g) and NHTO (0.17 ± 0.02 mg/100 g) when compared with the control (1.00 ± 0.17 mg/100 g) (Fig. 3).

### 3.4 Ethyl acetate fraction of Telfairia occidentalis increased liver glucokinase activity

**Fig. 4:**
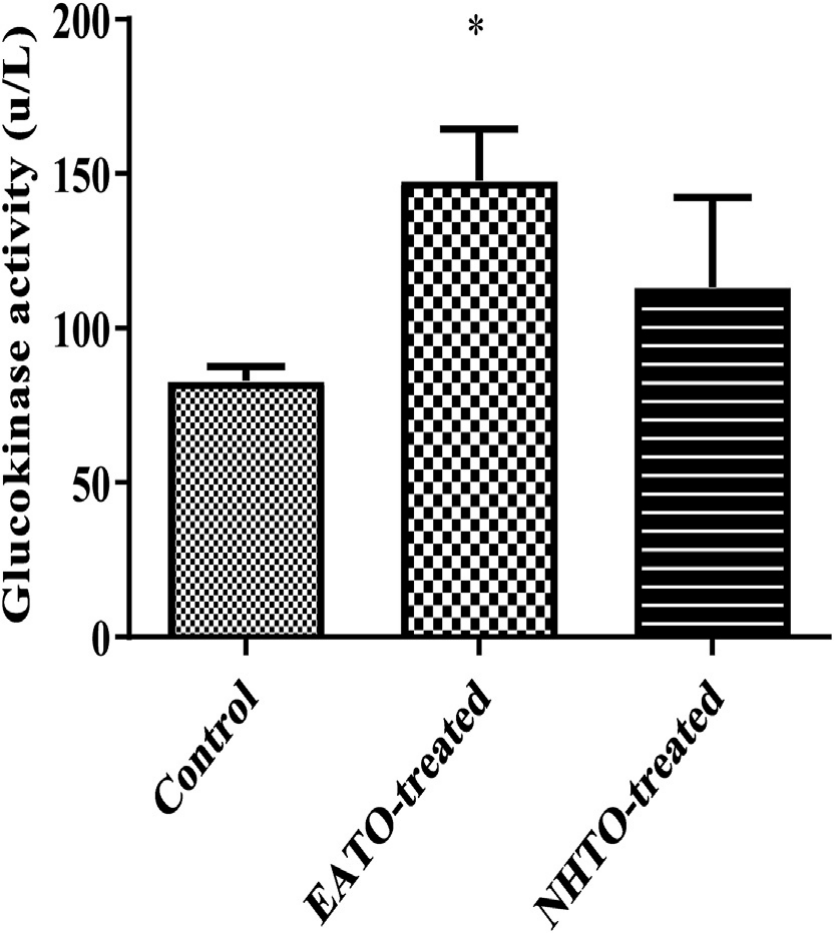
**Effects of fractions of *Telfairia occidentalis* on hepatic glucokinase activity.** ∗P < 0.05 vs control. EATO: Ethyl acetate TO fraction; NHTO; N-hexane TO fraction.

EATO caused a significant (p < 0.05) increase in liver glucokinase (from 82.90 ± 4.61 U/L to 147.80 ± 16.67U/L), while NHTO (113.3 ± 29.00 U/L) did not (Fig. 4).

### 3.5 NHTO reduced liver glucose-6-phosphatase activity

**Fig. 5:**
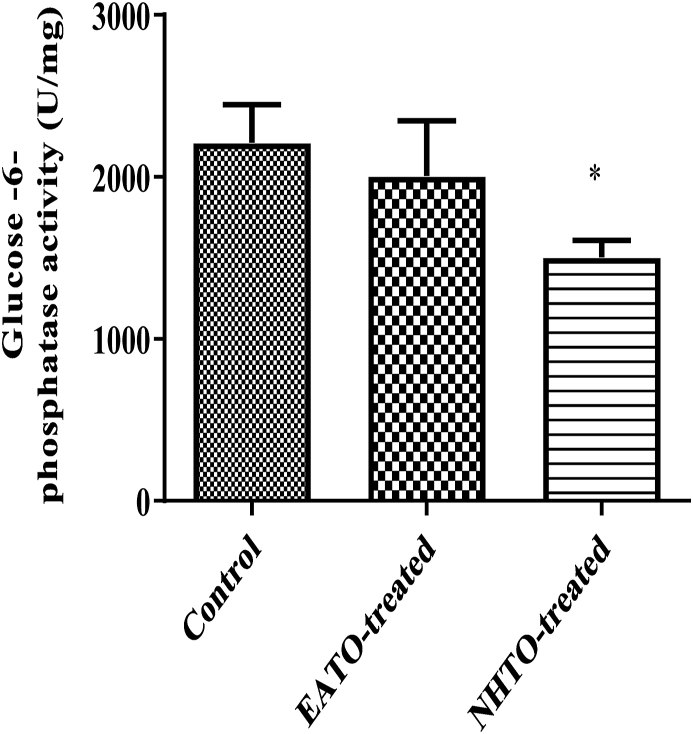
Effects of fractions of *Telfairia occidentalis* on Glucose-6-phosphatase activity. ∗p < 0.05. EATO: Ethyl acetate TO fraction; NHTO; N-hexane TO fraction.

NHTO significantly (p < 0.05) reduced hepatic G6Pase activity (from 2210.00 ± 236.00 U/mg to 1505.00 ± 103.30 U/mg), while EATO (2006 ± 340 U/mg) did not (Fig. 5).

### 3.6 EATO and NHTO had no significant effect on liver LDH activity

**Fig. 6:**
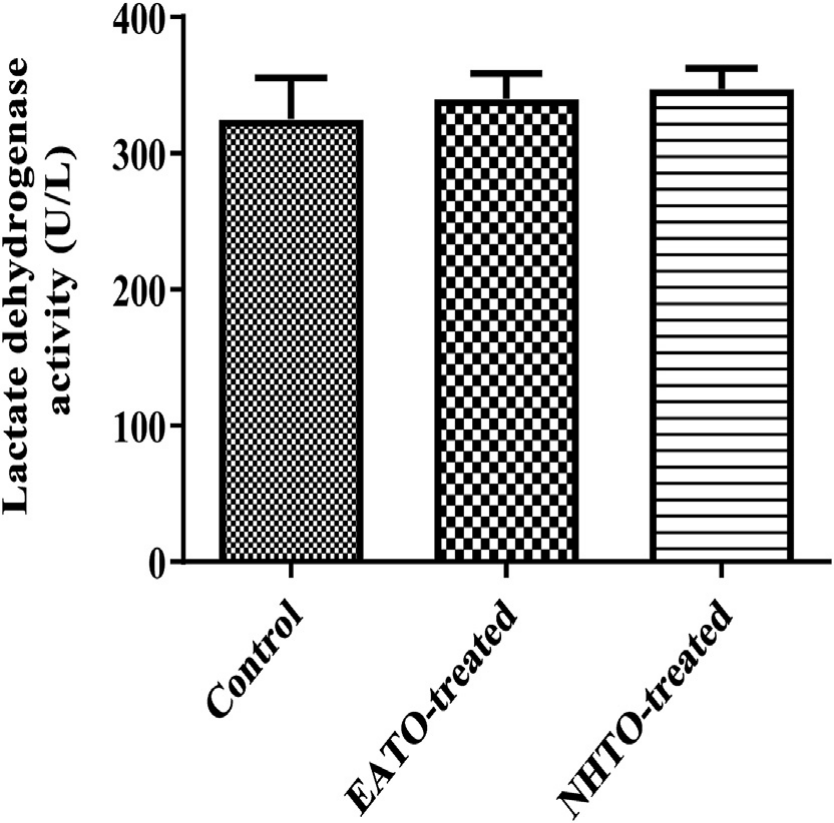
**Effects of fractions of *Telfairia occidentalis* on lactate dehydrogenase activity.** EATO: Ethyl acetate TO fraction; NHTO: N-hexane TO fraction.

Neither EATO (340.00 ± 18.56 U/L) nor NHTO (347.30 ± 15.05 U/L) caused any significant change in the liver LDH activity when compared to the control (325.30 ± 30.22 U/L) (Fig. 6).

### 3.7 EATO and NHTO had no significant effect on G6Pconcentration

**Fig. 7:**
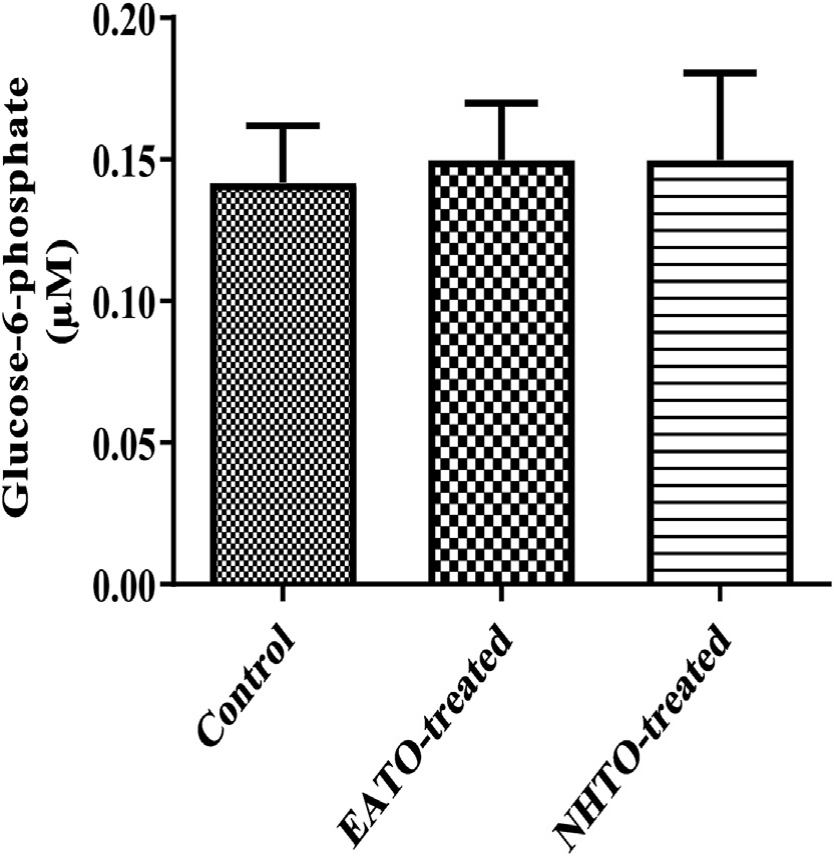
**Effects of fractions of *Telfairia occidentalis* on plasma glucose-6-phosphate**. EATO: Ethyl acetate TO fraction; NHTO; N-hexane TO fraction.

Neither EATO (0.15 ± 0.02 μM) nor NHTO (0.15 ± 0.03 μM) caused any significant change in the liver G6Pconcentration when compared to the control (0.14 ± 0.02 μM) (Fig. 7).

### 3.8 EATO and NHTO increased plasma pyruvate concentration

**Fig. 8:**
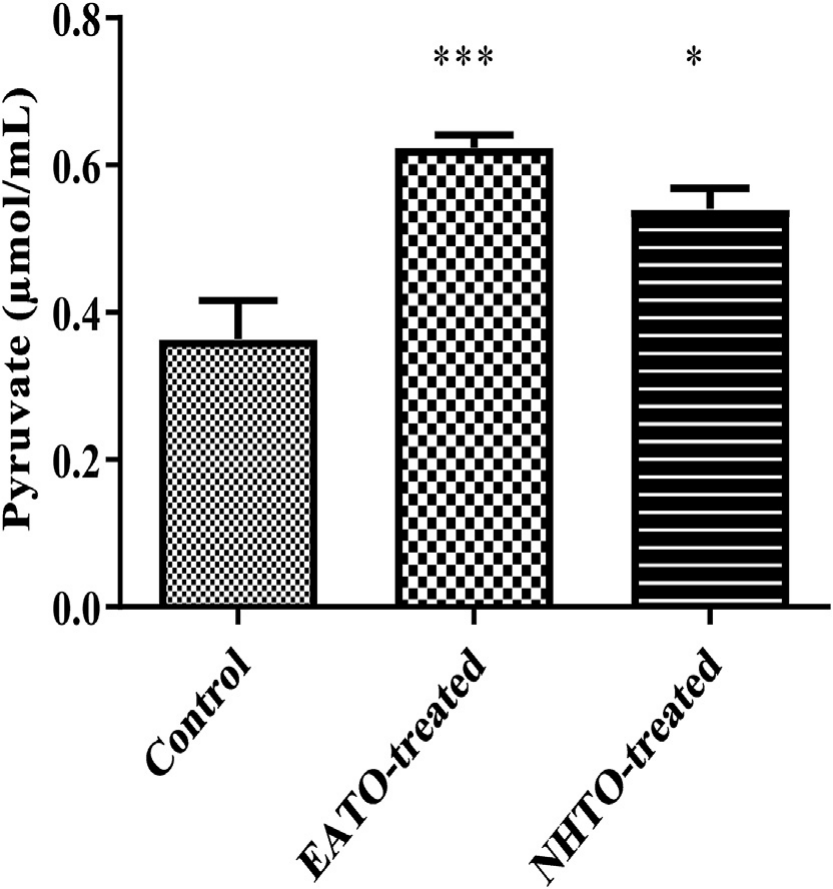
**Effects of fractions of *Telfairia occidentalis* on plasma pyruvate.** ∗∗∗P < 0.001; ∗P < 0.01 vs control. EATO: Ethyl acetate TO fraction; NHTO; N-hexane TO fraction.

Both EATO (p < 0.001) and NHTO (p < 0.01) significantly increased the pyruvate concentration from 0.36 ± 0.05 μmol/ml to 0.62 ± 0.02 μmol/ml and 0.54 ± 0.03 μmol/ml respectively (Fig. 8).

### 3.9 EATO and NHTO had no significant effect on plasma lactate concentration

**Fig. 9:**
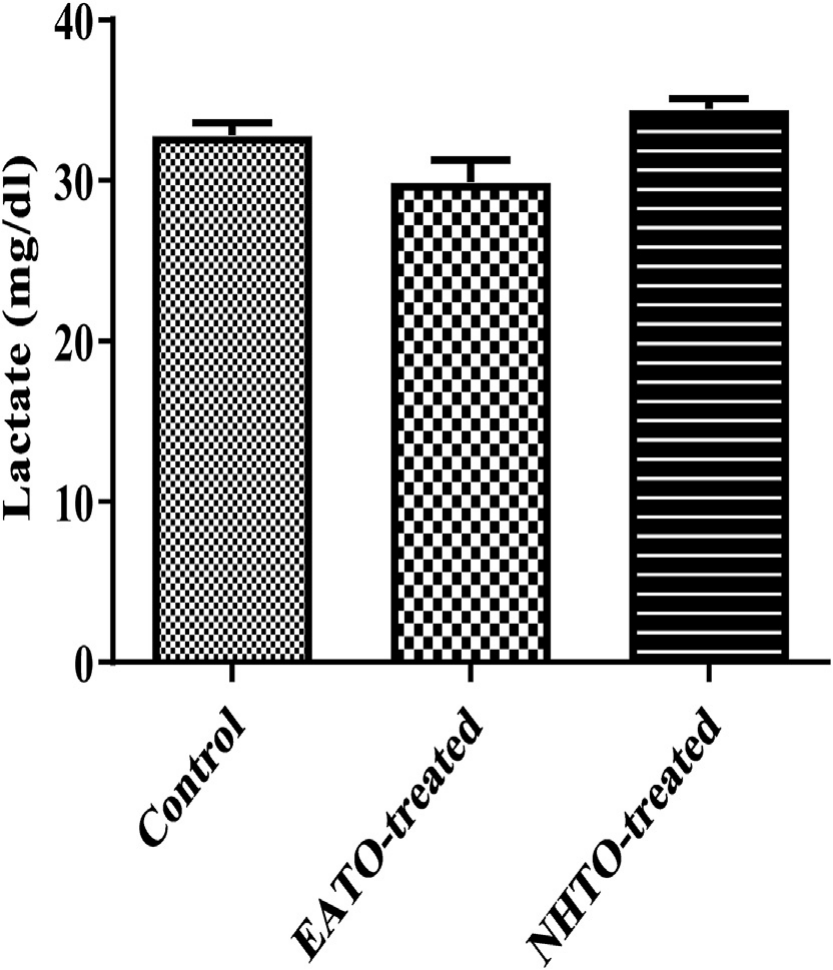
Effects of fractions of *Telfairia occidentalis* on plasma lactate. EATO: Ethyl acetate TO fraction; NHTO; N-hexane TO fraction.

Neither EATO (32.83 ± 0.75 mg/dl) nor NHTO (34.44 ± 0.64 mg/dl) caused any significant change in the lactate concentration when compared to the control (32.83 ± 0.75 mg/dl) (Fig. 9).

## 4 Discussion

N-hexane and ethyl acetate are solvents of different polarities being used in biological experiments to study plant bioactive components’ effects because they can dissolve different plant bioactive components [16,17]. In this research, n-hexane and ethyl acetate fractions of TO extract were used to investigate the cellular fate of glucose following hypoglycaemia caused by TO-mediated insulin increase. Then, we showed which of the two fractions contains a higher hypoglycaemic component of TO. Some biochemical parameters in the glucose metabolic pathway were determined following administration of ethyl acetate TO (EATO) and n-hexane TO (NHTO) fractions.

Consistent with previous findings [12,14], the present study showed a significantly increased plasma insulin and decreased glucose in the rats treated with EATO, affirming insulin’s involvement in the hypoglycaemic effect of TO. The hypoglycaemic effect of insulin has been well-documented [[21], [22], [23], [24]]. Besides, a single 250 mg/kg dose of EATO had a significant hypoglycaemic effect in diabetic rats [9].

The homeostasis of glucose is carried out by two signalling cascades: insulin-mediated glucose uptake (IMGU) and glucose-stimulated insulin secretion (GSIS). The IMGU cascade allows insulin to increase glucose uptake by body tissues, primarily skeletal muscle and adipose tissue via glucose transporter-4 (GLUT-4) [25,26], and the liver, β-cells, kidney and intestine via GLUT-2, while suppressing glucose generation by hepatic cells [27]. When blood glucose increases, such as after a meal, activation of the insulin cascade’s downstream signalling begins when insulin extracellularly interacts with the tissue’s insulin receptor and culminates with glucose transport/uptake from the blood into the tissue cells. The glucose transported into tissue cells undergoes three major cellular utilisation pathways including conversion to pyruvate for further oxidation via aerobic and anaerobic glycolysis, storage as glycogen for later use, and conversion to other metabolites to be used in other biosynthetic and metabolic pathways [28]. Under anaerobic conditions, glycolysis and pyruvate used for ATP production generate lactate as a by-product in body tissues, especially in skeletal muscle. The lactate can be used by muscles to restore glycogen after intense exercise, converted back to pyruvate and oxidised in the tricarboxylic acid cycle (TCA) under aerobic glycolysis, or converted back to glucose via gluconeogenesis [29,30].

Liver glucokinase (GCK) activity was also significantly increased by EATO fraction in this research. GCK is an enzyme that is central to liver glucose regulation during fasting and after meals. When blood glucose increases after a meal, the excess glucose is transported into the liver. In the liver, glucose-6 phosphate (G6P) is formed when GCK phosphorylates glucose. The G6P formation is the first step in glycogen synthesis and glycolysis via pyruvate. GCK converts glucose to G6P in hepatocytes and serves as a glucose sensor in pancreatic β-cells and blood glucose regulating neuroendocrine cells of the gut and brain [31]. Mutations of the GCK gene can cause unusual forms of diabetes or hypoglycaemia [32]. Liver GCK is primarily upregulated by insulin [33]. Taken together, the increased liver GCK activity and plasma insulin agree with the report that GCK activity is increased by insulin in the liver and by glucose in pancreatic β-cells [34,35].

Interestingly, liver glycogen decreased in all the treated groups rather than the expected increase. On the contrary, pyruvate significantly increased in both EATO and NHTO-treated groups, while lactate did not change significantly compared to control. Because the fate of glucose taken up by cells is either converted to pyruvate/lactate and oxidised or stored as glycogen; and there was no increase in the concentrations of both glycogen and lactate while pyruvate concentration and liver glucokinase activity increased in the EATO group, it is reasonable to conclude that glucokinase initiated the phosphorylation of glucose leading to its conversion to G6P and subsequent conversion to pyruvate in the glycolytic pathway. The absence of any significant change in the G6P and lactate concentrations in the EATO group suggests that most of the G6P formed due to the action of glucokinase proceeded in the glycolytic pathway to form pyruvate. This is consistent with the insignificant change in hepatic lactate dehydrogenase (LDH) activity and lactate concentration. Similarly, in the NHTO group, the insignificant increase in plasma insulin caused insignificant reduction in plasma glucose and an insignificant increase in GCK activity but a significant increase in pyruvate concentration. The significant increase in pyruvate despite the insignificant increase in GCK was probably due to the significant contribution of glycogenolysis to the G6P pool. Thus, while both glucose and glycogenolysis contributed to the G6P pool in EATO, glycogenolysis alone was mainly responsible for the G6P in NHTO.

Taken together, the increase in plasma insulin in EATO resulted in reduction in plasma glucose and activation of GCK to convert glucose to G6P and finally pyruvate in the glycolytic pathway while little or no G6P could have been formed through GCK in NHTO. The reduction in plasma glucose shut the pathway of glycogenesis (because both hyperglycaemia and hyperinsulinaemia are necessary for glycogenesis); thus leading to glycogenolysis (counter-regulation). Counter-regulation characterised by glycogenolysis and hyperglucagonaemia had earlier been reported following TO-induced hypoglycaemia [15]. However, the G6P formed from glycogenolysis also had to proceed in the glycolytic pathway to end up as pyruvate due to the persisting hyperinsulinaemia and suppression of G6Pase activity. G6Pase is an enzyme, mainly expressed in the liver and kidney, which is important in breaking down G6P back to glucose and phosphate so that the glucose can be released back to the blood during starvation. The G6P that will be converted back to glucose by G6Pase is mainly produced from glycogenolysis and gluconeogenesis that occur during starvation [36]. It is inhibited by insulin and glucose, which are both high after meals, thereby reducing endogenous glucose production [37,38]. Thus, the present observation of an increase in glycolysis as evidenced by the increased pyruvate production following hepatic G6Pase suppression is consistent with the report that glucose-6-phosphatase over-expression lowers G6P and inhibits glycogen synthesis and glycolysis in hepatocytes [39].

Thus, this study showed that TO caused hypoglycaemia and increased the G6P pool for pyruvate production via insulin-dependent mechanism (insulin activation of GCK) in the EATO group and insulin-independent mechanism (via glycogenolysis) in the NHTO group. A critical look at the G6P data showed that the values in EATO and NHTO were slightly higher but not significantly different from control. This suggests that there were actual increases in G6P concentration in the treated groups, but the increases were probably masked by the rapid conversion of G6P to pyruvate. The present study is limited by the absence of data on the activities of glycogen phosphorylase, phosphoglucomutase and adenosine monophosphate-activated protein kinase (AMPK); the availability of which would have provided a better understanding of the events leading to glycogenolysis. Our study is also limited by lack of data on phosphofructokinase and pyruvate kinase, which would have strengthened our contention that G6P remained in the glycolytic pathway and proceeded to form pyruvate.

In general, higher hypoglycaemic and insulinotropic properties of EATO than NHTO could be due to the well-reported hypoglycaemic and antioxidant effects of phenolic compounds [40,41], which are higher in ethyl acetate plant fractions than in n-hexane fractions [16,17].

It is also noteworthy that the plasma level of lactate in the NHTO group was significantly higher than in the EATO group but not too different from the control level. If it had been higher than that of the control, could such an increase in lactate concentration have suggested a negative effect of TO? Lactate was mainly considered a dead-end product of glycolysis previously. However, because of the introduction of lactate shuttle [42], increased lactate production and concentration from anoxia or dysoxia appears to be the exception rather than the rule [43]. Lactate formation and subsequent distribution throughout the body have been described as the major mechanism whereby the coordination of intermediary metabolism can be accomplished in different tissues.

Ashford and Holmes [44] showed that the disappearance of lactate and oxygen consumption are correlated in the brain, indicating an aerobic utilisation of lactate by brain tissue. Although glucose derived from blood is the primary energy source for generating ATP in the brain, glycogen synthesised from glucose in astrocytes is an important energy reserve [45]. The astrocytic glycogen is then broken down through glycogenolysis/glycolysis to produce lactate as a neuronal energy substrate transported by monocarboxylate transporters (MCTs) [46]. Besides, the genetic/pharmacologic inhibitions of glycogenolysis and/or lactate transport impair neuronal survival under severe hypoglycaemia, as well as axon transmission and hippocampus-related memory formation [[47], [48], [49]]. Glycolytic lactate dehydrogenase (cLDH) converts pyruvate to lactate, and under aerobic conditions, lactate is the main substrate of the tricarboxylic acid (TCA) cycle and, as such, must be considered as the main molecule coupling between the glycolytic and the TCA cycle pathways, one in the cytosol and the other in the mitochondrion, respectively. Lactate is transported from the cytosol into the mitochondrion via a monocarboxylate transporter (MCT) [50,51], where it is oxidised to pyruvate by mitochondrial lactate dehydrogenase (mLDH) and also provides the mitochondrion with NADH. Thus, lactate can move into the TCA cycle like pyruvate as opposed to the earlier dogma that pyruvate is the only useful product of glycolysis that can enter the TCA cycle while lactate is just a waste product.

## 5 Conclusion

The present study showed that insulin-mediated TO-induced hypoglycaemia resulted in the stimulation of glycolysis and pyruvate production via insulin-dependent and insulin-independent mechanisms.

## Declarations of interest

The authors declare that they have no known competing financial interests or personal relationships that could have appeared to influence the work reported in this paper.

## Funding

This research did not receive any specific grant from funding agencies in the public, commercial, or not-for-profit sectors.

## CRediT author statement

Toyin Mohammed Salman: Conceptualisation, Research supervision, Data interpretation, Manuscript drafting. Sheu Oluwadare Sulaiman and Abdul-Musawwir Alli-oluwafuyi: Conceptualisation, Data analysis, Data interpretation, Manuscript drafting. Mayowa Adewale Iyanda: Carried out the research, Data analysis, Data interpretation, Manuscript drafting. Abdullateef Isiaka Alagbonsi: Data interpretation, Manuscript drafting and revision. All authors read and approved the final manuscript to be published.

## Data statement

The data that support the findings of this study are available from the corresponding author, [Sheu Oluwadare Sulaiman], upon reasonable request.

## CRediT authorship contribution statement

**Toyin Mohammed Salman:** Conceptualization, Research, Supervision, Data curation, Writing – original draft, interpretation. **Mayowa Adewale Iyanda:** Carried out the research, Data curation, Formal analysis, Writing – original draft, Data interpretation. **Abdul-Musawwir Alli-oluwafuyi:** Writing – original draft, Conceptuzlisation, Data curation, Formal analysis, Data interpretation. **Sheu Oluwadare Sulaiman:** Writing – original draft, Conceptualization, Data curation, Formal analysis, Data interpretation. **Abdullateef Isiaka Alagbonsi:** Data curation, Writing – original draft, Data interpretation. All authors read and approved the final manuscript to be published.
